# Framework, method and case study for the calculation of end of life for HWL and parameter sensitivity analysis

**DOI:** 10.1038/s41598-020-72514-5

**Published:** 2020-11-11

**Authors:** Rui Xiang, Jing-Cai Liu, Ya Xu, Yu-Qiang Liu, Chang-xin Nai, Lu Dong, Qi-Fei Huang

**Affiliations:** 1 State Key Laboratory of Environmental Benchmarks and Risk Assessment, Chinese Research Academy of Environment Sciences, Beijing, 100012 China; 2 Research Institute of Solid Waste Management, Chinese Research Academy of Environment Sciences, Beijing, 100012 China; 3 College of Water Science, Beijing Normal University, Beijing, 100875 China

**Keywords:** Environmental chemistry, Environmental impact, Hydrology

## Abstract

Mass construction and operation of hazardous waste landfill infrastructure has greatly improved China’s waste management and environmental safety. However, the deterioration of engineering materials and the failure of landfill may lead to the release of untreated leachate rich in persistent toxic pollutants to the soil and shallow groundwater. Accordingly, we develop the framework and process model to predict landfill life by coupling the landfill hydrological performance model and material degradation model. We found that the decrease rate of the concentration of persistent pollutants in leachate was significantly slower than the deterioration rate of the landfill engineering materials. As a result, when the materials failed, the leachate with high concentrations of persistent pollutants continued to leak, resulting in the pollutants concentration in surrounding groundwater exceeding the acceptable concentration at around 385 a, which is the average life of a landfill. Further simulation indicated that hydrogeological conditions and the initial concentration of leachate will affect landfill lifespan. The correlation coefficients of concentration, the thickness of vadose zone and the thickness of aquifer are − 0.79, 0.99 and 0.72 respectively, so the thickness of vadose zone having the greatest impact on the life of a landfill. The results presented herein indicate hazardous waste landfill infrastructure reinvestment should be directed toward long-term monitoring and maintenance, waste second-disposal, and site restoration.

## Introduction

Landfill represents one of the predominant methods for hazardous waste disposal^1^. For example, in Germany, approximately 21% of the 21.81 million tons of hazardous waste generated annually are landfilled. In the entire European Union (EU)^2^, the ratio of hazardous waste that is landfilled to total hazardous waste generated is even greater, approaching 40%. In developing countries like China, the use of landfill is more extensive, and this is still expanding because of its affordability and low technical barriers^3^.

The mass construction of hazardous waste landfills (HWL) may bring many hidden dangers, especially for developing countries, one of which is that HWL failure may cause serious environmental pollution and human health problems^4^. Like other materials, engineering materials such as high density polyethylene (HDPE) geomembrane and drainage pipe used in landfills also have life spans^5^. When these materials deteriorate and their performance degrades, the landfill will fail and reach the end of life (EOL)^6^. However, in contrast to the degradation of engineering materials, the hazardous component of hazardous waste landfill is heavy metal and its degrades slowly behavior is very weak, so it will continue to cause harm for a long time. When the landfill fails, these persistent harmful components (PHCs) are released from the solid phase and leak into the soil, groundwater, and other environmental media together with the leachate, where they cause great harm to the ecological environment and human health^7^.

This has increased the necessity to predict lifespan for the duration from the operation of hazardous waste landfills to their EOL. The lifespan prediction, if accurately conducted, provides a buffer to landfill failure (such as waste removal or other engineering measures to extend landfill life) and prevent the catastrophic environmental consequences caused by its failure^8^. However, few studies of the EOL of landfills have been conducted because HWL have mistakenly been regarded as a once and for all disposal method, with an infinite lifespan^9,10^. Indeed, almost all countries have regulations that regulate the time frame of post-closure care for hazardous waste landfill facilities, but these are only related to the operation time associated with the storage capacity of the landfill, not the service life or physical life related to its durability. In addition, some countries suggest (such as the United States) or force (such as China in its latest national standards) the life length of all hazardous waste landfill facilities to be predicted when they are designed, but no specific methods or procedures for doing this are provided. Moreover, while some methodologies have been used to assess the service life of landfill components or materials^11^, none have been applied to entire landfills. For example, Xu et al.^12^ studied the rapid decrease of permeability of guide discharge medium in hazardous waste landfill caused by silting and evaluated its service life. Liu et al.^11^ investigated the law of oxidative aging of HDPE and the main influencing factors through experiments, and then developed a method for calculation of the half-life of HDPE on this basis. Other studies investigated the long-term effects of solidified materials on solidifying and stabilizing harmful ingredients in solid waste (leachate)^13^.

To make up for the deficiencies mentioned above, in this study, a framework and method for life prediction of hazardous waste landfills was developed, then applied to predict the life of a typical hazardous waste landfill. The results presented herein will provide insights into the durability lifespan of hazardous waste landfills, as well as the long-term environmental risks associated with them. It is expected to provide theoretical support for life cycle assessment, long-term risk management and control, and design optimization based on life cycle of hazardous waste landfill facilities.

## Material and methods

### Framework for design life calculation

#### End of life index and its threshold

In this paper, the term ‘design life’ is defined as the period during which the HWL can ensure that the surrounding groundwater is free from contamination of landfilled waste. Therefore, the concentration of pollutants in surrounding groundwater was selected as the end-of-life index. The EOL is achieved when the groundwater quality becomes unacceptable because of the increasing leachate leakage rate as the HWL performance degrades^14^.

**Figure 1:**
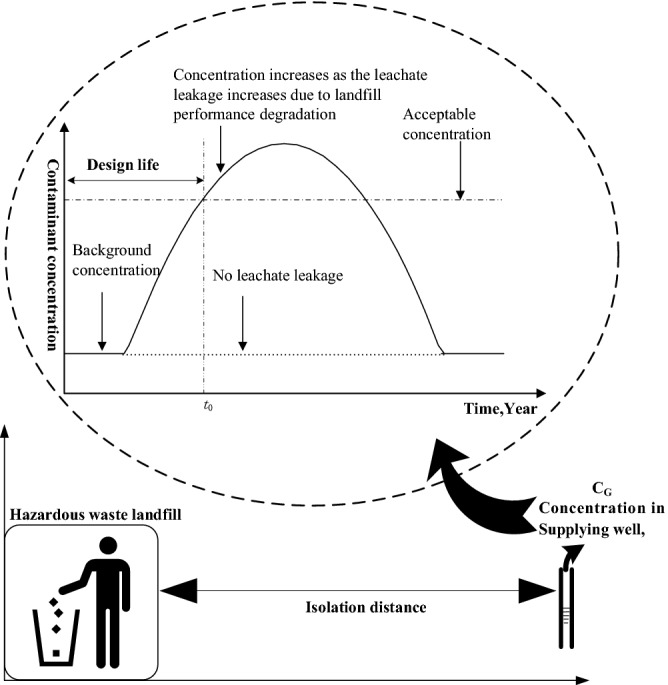
Framework for calculation of HWL design life.

If the process of changes in groundwater pollutant concentrations under landfill aging conditions can be simulated and acceptable groundwater quality (AGQ) can be determined, then the design life can be determined according to the framework shown in Fig. 1. The following describes how to determine AGQ and simulate evolution of groundwater concentration with consideration of the aging process of the landfill’s main functional units and materials.

#### Acceptable groundwater quality

A method for determining drinking water standards based on acceptable risks is recommended by the WHO^15^. This method is more functional as it determines acceptable concentration limits in groundwater based on habits (e.g., frequency and quantity of drinking), physical characteristics of the exposed population (e.g., lifespan, weight, and age), and toxicity of the target pollutant. The method based on the dynamic update of population exposure and toxicological parameters of pollutants will continue to improve. Therefore, this paper adopted this risk-based method to determine the acceptable contaminant concentrations in groundwater. The risk of leachate pollution to drinking groundwater was characterized by the hazard quotient (HQ) for non-carcinogenic hazards and the cancer risk for carcinogenic hazards. HQ was calculated using Eq. (1)^16^:

1 $${\text{HQ}} = \frac{CDI}{{RfD}}$$

2 $${\text{CDI}} = \frac{{c_{g} \times I_{w} }}{{w_{b} }}$$

The cancer risk (CR) was estimated using the formula as follows^7^:

3 $$\left\{ {\begin{array}{*{20}l} {Risk = CDI \cdot SFRisk \le 0.01} \hfill \\ {Risk = 1 - \exp ( - CDI \cdot SF)Risk > 0.01} \hfill \\ \end{array} } \right.$$

Generally, when the value of HQ and/or risk is < 1, the health risk is acceptable. Therefore, the AGQ can be calculated by solving Eq. (1) and Eq. (2) or Eq. (1) and Eq. (3).( See Table 1 for parameter definition and unit).

**Table 1:** The meanings and units of parameters in all formulas.

Symbol	Parameter	Unit
RfD	Reference dose for toxicity of target contaminants	mg/kg/day
CDI	Average daily intake	mg/kg/day
Cg	Concentration of targeted contaminants in groundwater	μg/L
I_w_	Average water consumption	L/day
W_b_	Average body weight	kg
SF	Cancer slope factor for target contaminants	mg/kg/day^−1^
K_gm_	the permeability coefficient of HDPE membrane	cm/s
N	Hole number	per/hm^2^
N_0_	Hole number in the initial period	per/hm^2^
K	Hydraulic conductivity of the drainage layer	cm/s
Kd_0_	Initial conductivity	cm/s
K_waste_	Conductivity of the waste immediately above the drainage layer	cm/s
C_t_	Contaminant concentration in the leachate	mg/L
C_0_	Initial concentration in the leachate	mg/L
t	Time	year
R	Liquid solid ratio	l/kg
k	A species- and waste-specific constant	kg/l
a	Specific constants	kg/l
b	Specific constants	kg/l
x	x coordinate	m
c	Contaminant concentration at distance x	mg/L
v	The velocity of Groundwater	m/s
γ	First order decay rate	s^−1^
n	The effective porosity	–
DL	Longitudinal coefficient of hydrodynamic dispersion	m^2^/s
α	Medium's dispersivity	m
Dm	Coefficient of molecular diffusion	m^2^/s

### Contaminant concentration simulation under aging conditions

Groundwater pollution around the landfill site was caused by leachate leakage through geomembrane defects^17^. Ideally, without considering the aging of the main functional units of the landfill, the annual production and leakage of leachate can be regarded as approximately stable^18^. In this condition, provided that the surrounding groundwater flow is stable, the contaminant concentrations in groundwater will reach a dynamic equilibrium due to the balance between leachate leakage and regional groundwater recharge^19^. However, if the production and leakage of leachate continues to increase due to the aging of the landfill’s main units, the concentrations of contaminants in groundwater will continue to increase and ultimately outnumber the AGQ and reach its EOL.

Therefore, in order to simulate the aggravation of groundwater pollution caused by the degradation of landfill performance, three processes must be considered: the process of leachate generation and leakage, the process of performance degradation, and the process of leakage and migration in groundwater. In this study, three modules were introduced to account for the three processes. The Hydrologic Evaluation of Landfill Performance (HELP)^20^ was used to predict the generation and leakage of leachate; the Degradation Model for Functional Units (DMFU)^21^ module was applied to estimate the aging of main functional units, and the Environmental Protection Agency Composite Model for Leachate Migration with Transformation Products (EPACMTP)^22^ was used to simulate the groundwater pollution process under leachate leakage conditions.

**Figure 2:**
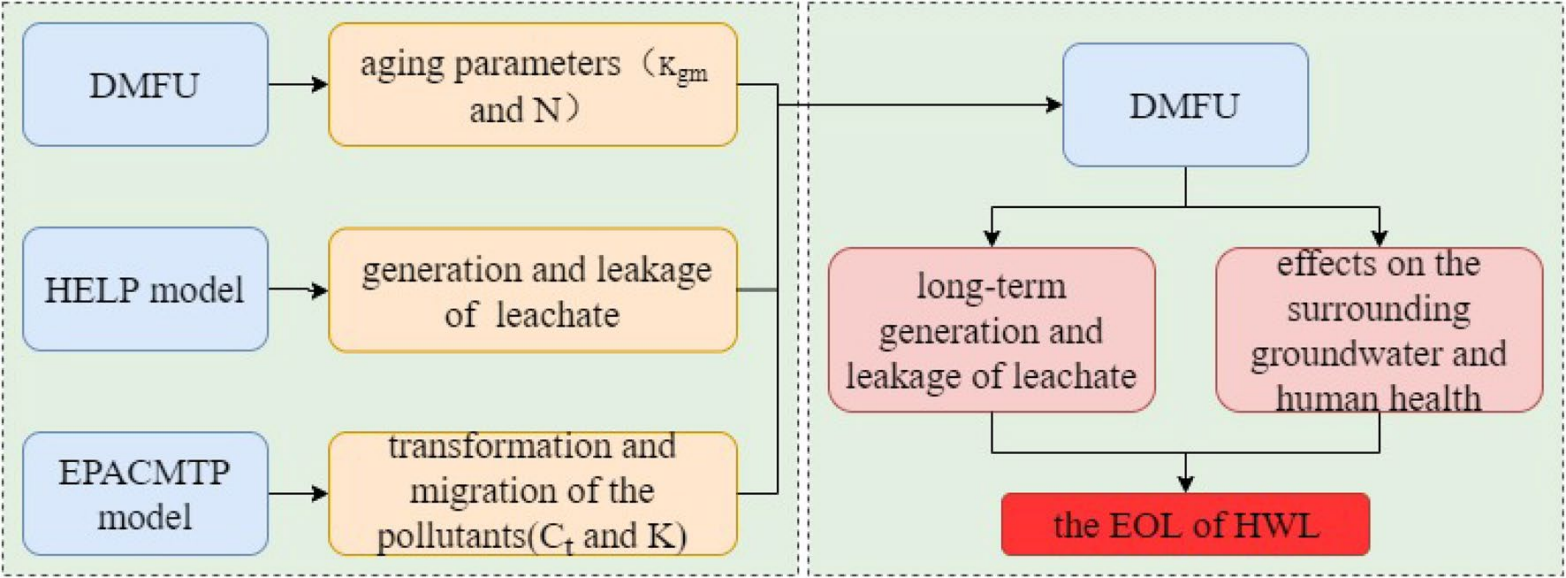
Models, parameters and their coupling processes.

The three modules were connected in sequence. First, aging parameters were calculated by the DMFU module, then the generation and leakage of landfill leachate were predicted using the HELP model, and finally the transformation and migration of the pollutants in the subsurface mediums were simulated by the EPACMTP module. In the simulation process, the solution process was applied to simulate the generation, discharge, and migration of leachate in the underground medium, as well as in each time step. It should be noted that in the whole process, the basic principles and equations of the HELP and EPACMTP software were used rather than the software itself. Additionally, the DMFU model was used to study the long-term generation and leakage of leachate, as well as its effects on the surrounding groundwater and human health (Fig. 2).

#### Hydrological evaluation of landfill performance (HELP) model

The HELP model was created by the U.S. Army Corps of Engineers Waterways Experiment Station for the United States Environmental Protection Agency (USEPA)^23^. This model is used in the design and management of landfills around the world as it accepts weather, soil, and design data and uses solution techniques that take into account the effects of rainfall, runoff, infiltration, evapotranspiration, lateral subsurface drainage, leachate recirculation, unsaturated vertical drainage, and leakage through soils, geomembranes, or composite liners, which facilitates rapid estimation of the amounts of infiltration, drainage, leachate collection, and liner leakage^24^. The hydrological outcomes simulated by the HELP model included time series data, such as precipitation, evapotranspiration, and leakage. Consequently, this study used the HELP model to simulate the generation and leakage of leachate in HWL^25^.

#### Degradation model for functional units (DMFU)

The aging of drainage media used in rainwater and leachate drainage systems is caused by clogging, which leads to decreased hydraulic conductivity (K_d_) and declines in drainage capacity^26^. The aging of HDPE membranes usually results from polymer oxidation and leads to increases in the permeability coefficient (*K*_gm_) and hole density (*N*), as well as ultimately increases leakage^27^. The three sub-modules were used to predict changes in the three parameters (i.e., the conductivity of drainage medium, permeability coefficient, and hole density of HDPE geomembrane) with time under aging conditions. Their outputs were used as the input in the HELP model to calculate the long-term generation and discharge of leachate.

A previous study^28^ demonstrated that under the influence of ultraviolet radiation and leachate corrosion, the permeability coefficient of the HDPE membrane will raise over 250–1000 years, and as aging continues, its impermeability will eventually be the same as its underlying clay (1 × 10^−7^ cm/s). Thus, the changes in *K*_gm_ with time can be expressed as follows:

4 $$K_{gm} (t) = 1.33 \times 10^{ - 10} \times (t - 250)$$

Drury^29^ assumed that due to the degradation of mechanical properties caused by oxidative aging, the number of holes in the geomembrane will double over a certain period. In a separate study^30^, it was reported that the number of holes in the geomembrane increased after 100 years, which doubled every 250 years. Thus, the changes in *N* over time can be expressed as a power function based on its initial value as follows^7^:

5 $$N(t) = N_{0}^{0.004(t - 100) + 1}$$

Clogging of the drainage layer will cause the hydraulic conductivity (K_d_) of leachate collection and drainage system (LCDS) to decrease. Because clogging progress is affected by many elements, including the concentration of leachate, and due to the biochemical characteristics of the clog and the size and distribution of drainage pebbles, it is particularly complex. Consequently, it is difficult to accurately describe the changes in K_d_ over time. A simplified model^31^ was adopted in this study as follows:

6 $${\text{K}}_{{\text{d}}} ({\text{t}}) = \left\{ {\begin{array}{*{20}l} {{\text{e}}^{ - 0.0375t} \times K_{d0} (K_{d} \ge K_{waste} )} \hfill \\ {K_{waste} \;\;(K_{d} = K_{waste} )} \hfill \\ \end{array} } \right.$$

The degradation of the LCDS may also include failure of the drainage pipe, which will cause an increase in leachate drainage distance and leachate levels in the LCDS. The feasibility of pipe failure can be estimated using Eq. (4)^29^:

7 $$p(X = k) = \frac{{e^{\lambda } \lambda^{k} }}{k!}$$

where p (X = k) is the probability of observing k pipes in failure, and k takes values of 0, 1, 2, …; λ is the failure rate of 1 pipe.

#### EPA composite model for leachate migration with transformation products

EPACMTP was developed by HydroGeoLogic, Inc. (HGL) for the USEPA to simulate the effects of the release of chemical components in SW from landfills. Currently, it is procurable from the EPA’s website (Version 2.0). The EPACMTP model^32^ supplies a multimedia tool to simulate chemical transport in the underground media through analytical solutions and numerical methods. Furthermore, it also takes into account the uncertainty of parameters in the estimation of exposure concentrations using the Monte Carlo model.

Leachate concentration is the mass of dissolved constituents per unit volume of water emanating from the base of the landfill. This parameter provides the upper boundary condition for the EPACMTP simulation of constituent fate and transport through the vadose and aquifer zone. The EPACMTP provides three choices for simulating the change of leachate concentration, which is a continuous, pulse, or depleting source. Generally, the composition and concentration of leachate will decrease over the lifecycle of the landfill. One reason for the change in leachate concentrations is that the liquid (i.e., rainwater and groundwater) penetrating the landfill will constantly clean the SW. Thus, in this study, the consumption source model^12^ was used to describe this process:

8 $$C_{t} = C_{0} e^{ - KR}$$

9 $${\text{K}} = {\text{m}}\ln \left( {C_{0} } \right) + {\text{c}}$$

The EPACMTP supplies a variety of choices for simulating the movement of pollutants in leachate in the aquifer under different hydrogeological conditions. For a single unconfined aquifer with a uniform thickness, assuming that the saturated, porous medium properties are isotropic and homogeneous, and that the regional velocity field in the aquifer is constant over time, uniform at all points, and unidirectional in the positive x-direction, the contaminant migration through an impacted groundwater system resulting from the transport processes associated with the effect of groundwater flow (i.e., advection) and the effects of attenuation processes^33^, such as degradation, dispersion, and retardation, can be characterized by the following equations^12^:

10 $$\frac{\partial c}{{\partial t}} = D_{L} \frac{{\partial^{2} c}}{{\partial x^{2} }} - \frac{x}{n}\frac{\partial c}{{\partial x}} - R\gamma c,$$

11 $$D_{L} = \alpha v + D_{m} .$$

The solutions of the above advection–dispersion equations have been reported for different boundary conditions. When the equations exhibit an upper boundary of an exponentially declining source (see Eq. (5), it can be solved with the Laplace transformation method and the analytical solution as follows^29^:

12 $$\frac{c(x,t)}{{c_{d} }} = \frac{1}{2}e^{ - \lambda t} \left\{ {{\text{e}}^{{\left[ {\frac{vx}{{2D_{L} }}(1 - \sqrt \mu )} \right]{\text{erfc}}\left[ {\frac{x - vt\sqrt \mu }{{2\sqrt {D_{L} t} }}} \right]}} + {\text{e}}^{{\left[ {\frac{vx}{{2D_{L} }}(1 + \sqrt \mu )} \right]{\text{erfc}}\left[ {\frac{x + vt\sqrt \mu }{{2\sqrt {D_{L} t} }}} \right]}} } \right\}$$

13 $$\upmu = 1 + \frac{4(\gamma - \lambda )D}{{v^{2} }}.$$

Equations (9) and (10) are applicable to the contaminant transportation in both vadose and aquifer systems. When applied to the simulation of aquifer flow, the transformed output from the previous vadose pathway forms the input for the next aquifer. For more details, see Drury^29^.

### Case study

#### Site description

In this study, an HWL in Southeastern China was used as a research case. It covers an area of 2.5 hm^2^ and has a design volume of 27.4 million m^3^. According to the Standard for Pollution Control on the Security Landfill Site for Hazardous Wastes^34^ by the Ministry of Ecological Environment, the distance of groundwater wells around the landfill site should not be less than 800 m; therefore, a groundwater well located 800 m downstream of the landfill was selected, and the contaminant concentration in this well was simulated and compared with the AGQ to determine whether the landfill reached its EOL.

The target aquifer was unconfined, with uniform thickness and an isotropic and homogeneous porous medium. Although there was a certain degree of fracture development in the underlying rocks^35^, there was almost no hydraulic connection with the target aquifer, which this study did not consider. Additionally, it was assumed that the regional velocity field in the aquifer was constant with time and uniform at all points^36^. Consequently, the groundwater flow could be calculated directly using Darcy’s law and inserted into the analytical solutions of the advection–dispersion equation with exponential decay boundary condition to forecast the pollutant concentration in the aquifer^37^.

A total of 13 pollutants were detected in the leachate. Of these, the pollutants whose concentrations were evidently lower than the concentration limits of groundwater quality standards^38^ were excluded and not considered in the following simulation. As a result, only Zn, F, and Cr remained; therefore, these were taken as the target pollutants. Then, it was assumed that the initial pollutant concentration of the three pollutants in the background groundwater was 0, and other sources (e.g., geogenesis) were excluded during the simulation period of 1000 years^39^.

#### Model input parameters

Model input parameters include the following categories: surface infiltration parameters, landfill design parameters, leachate concentration, aging model parameters, and porous media flow and solute transport parameters. The value of aging model parameters is shown in Eqs. (3) to (5), while the values of the other parameters are shown in Table 2.

**Table 2:** Summary of main input parameters.

Parameters			Value	Source
Surface infiltration parameters	Net rainfall (mm/y)		300	
	Land slope (%)		4	Actual survey
	Maximal slope length (m)		200	Actual survey
Landfill design parameters and leachate concentration	Bottom area (ha)		2.5	Measurement
	Final landfill height (m)		11	Actual survey
	^c^Pollutant concentration in the leachate (mg/L)	Zn	75	35
		Inorganic fluorides	100	35
		Cr	2.5	35
	Slope of primary drainage layer (%)		5	Actual survey
	Thickness of primary drainage layer (m)		0.3	Actual survey
	Initial conductivity of primary drainage layer (cm/s)		0.1	Actual survey
	Slope of secondary drainage layer (%)		5	Actual survey
	Thickness of secondary drainage layer (mm)		6.3	Actual survey
	^a^Conductivity of secondary drainage layer (cm/s)		0.1	Actual survey
	^b^Structure of liner system		Double artificial liner	Actual survey
	Hole density (#/hm^2^)		11	measurement
	Initial permeability of geomembrane (cm/s)		1E−13	Actual survey
	Thickness of clay base (m)		0.6	Actual survey
	Conductivity of clay base (cm/s)		1E−7	Actual survey
Porous media flow and solute transport parameters	Thickness of vadose zone (m)		13	Actual survey
	Vadose zone conductivity (cm/s)		5.79E−4	Actual survey
	Vadose zone longitudinal dispersivity (m)		0.6	Actual survey
	Aquifer thickness (m)		15	Actual survey
	Aquifer conductivity (cm/s)		2.66E−2	Actual survey
	Hydraulic gradient (%)		0.001	Actual survey
	Aquifer porosity		0.6	Actual survey
	Vertical dispersivity (m)		0.6	35
	Lateral dispersivity (m)		0.02	35

^a^after filtration by the primary drainage layer, clogging substances in the leachate decrease and the probability of the secondary drainage layer clogging was reduced to the minimal extent; therefore, clogging of the secondary drainage layer can be neglected and was not considered here.

^b^Because the secondary and primary liner systems use the same geomembrane, their aging processes were assumed to be the same; therefore, the aging parameters utilized were the same.

^c^The pollutant concentrations in leachate was assumed to be the control limit concentration specified by GB18598 for waste.

The relevant parameters listed in Table 3 were inserted into the equations and yielded threshold concentration concentrations of 2.35, 0.31, and 0.03 mg/L for Zn, F, and Cr in groundwater, respectively.

**Table 3:** Toxicity parameters of target contaminants.

Parameter	Zn	F	Cr
RfD_0_ (mg/kg/day)	0.300	0.004	0.500
SF_0_ (mg/kg/day)			0.003

## Results

**Figure 3:**
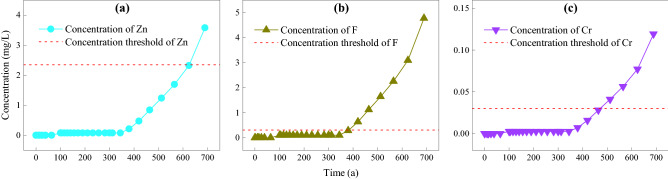
Computation result of EOL.

The EOL of HWLs based on different protective objects (contaminants) can be calculated using the three coupled models to calculate the concentration of pollutants in the groundwater wells at the safety protection distance, as well as the threshold concentration of different pollutants calculated in Sect. 2.3 (Fig. 3). The EOL of the landfill was 625 a based on preventing Zn from harming human health (Fig. 3a). As shown in Fig. 3b, the EOL of the landfill was 385 a based on preventing F from harming human health, while it was 470 a based on preventing Cr from harming human health. Therefore, the EOL is 385 a from the perspective of protecting human health because this was the lowest value among the three above elements.

**Figure 4:**
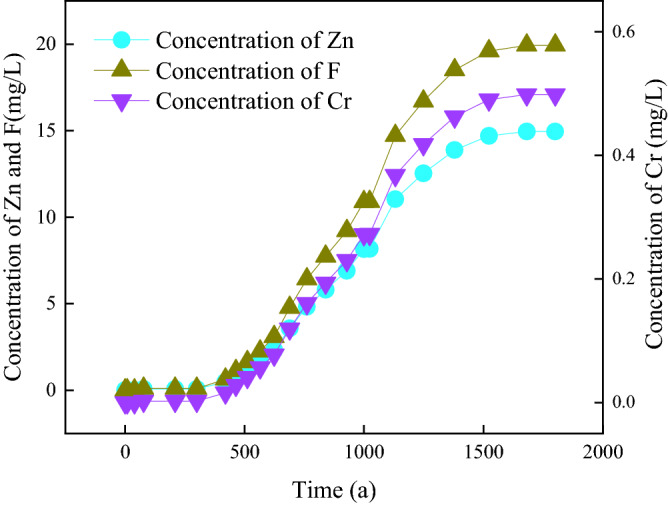
Duration of concentration in drinking well.

**Figure 5:**
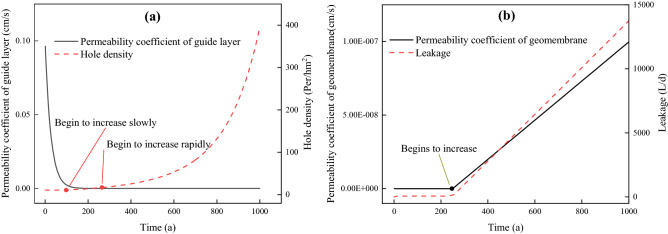
Duration of leachate leaking rate.

Landfills are determined to have reached their EOL based on whether the concentration of pollutants in drinking water exceeds a threshold concentration within a safe distance. The change in concentration of the three investigated pollutants (Zn, Cr, and inorganic fluoride) could be divided into three stages (Fig. 4). Taking Zn as an example, the first stage is the penetration time (BP) of pollutants from the reservoir bottom to the drinking well, which is when pollutants leak from the impervious layer, intercept pollution from the natural base layer and aeration zone, and diffuse through the aquifer to drinking wells. This stage is about 70 a. Stage 2 is when the concentration of Zn increases rapidly to a relatively stable level during the 70–330 a period. The rapid increase stage, which is about 70–78 a, is the period during which Zn reaches the drinking well and diffuses to equilibrium. From 78–330a, although adverse events occur such as an increase in the number of holes (100 a), an increase in the geomembrane permeability coefficient (250 a), and a decrease in the permeability coefficient of the drainage layer, the concentration of Zn remains relatively stable. The reason for this is as follows: according to Eq. (3), the vulnerability density increases exponentially; therefore, the increase in the initial period (100–250 a) is slower, while that in the later period (250 a) is faster (Fig. 5a). Simultaneously, after 250 a, the geomembrane permeability increases linearly (Fig. 5b), which causes the leakage to increase rapidly around 250 a (Fig. 5b). However, there is a lag time (approximately equal to the BP) before the concentration of pollutants in drinking wells responds to the rapid increase in leakage. This time is greatly affected by the thickness of the natural base layer and the vadose zone, as well as the groundwater flow rate^40,41^. In the present case, this time is about 70 a. After 330 a, the concentration of Zn starts to increase rapidly again, which continues to around 1500 a, at which point steady state is reached. The rapid increase in the concentration of Zn is because of the increase in hole numbers and the permeability coefficient of the geomembrane, as well as the decrease in the permeability coefficient of the drainage layer^42^. At about 1500 a, the impermeable membrane has completely aged, and the permeability coefficient of the drainage layer is no longer reduced; therefore, the concentration of Zn in drinking wells gradually tends to balance.

## Discussion

The EOL of HWLs is affected by many factors, such as the anti-aging properties of the material (geomembrane capacity and drainage layer anti-clogging ability) and the concentration of leachate and site factors (vadose zone thickness, permeability coefficient, aquifer thickness, and permeability coefficient)^40,43^. The aging properties of the material are often difficult to control, but the concentration of leachate can be controlled by managing the leaching concentration limits. Site factors can also be changed in the landfill site selection stage. The leaching concentration limits, thickness of the vadose zone, and aquifer thickness should be investigated to analyze the impact on the EOL of the landfill.

**Figure 6:**
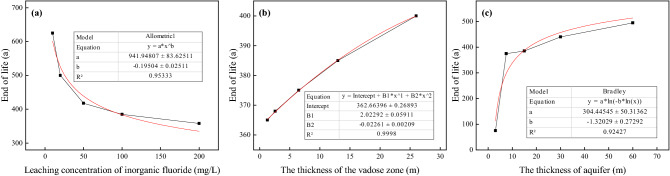
Correlation analysis of EOL relevant parameters.

Therefore, we kept the other parameters constant while changing the leaching concentration limits, thickness of the vadose zone and aquifer thickness to calculate the corresponding EOL and plot the EOL curve (Fig. 6). The relationship between the leaching concentration limits and EOL could be fitted into a power function curve with an R^2^ squared value of 0.95, indicating that the fitting result is reliable (Fig. 6a). According to the fitted curve, the EOL increases as the hazardous waste leaching concentration limit decreases. Moreover, correlation analysis of the leaching concentration limits and EOL showed that there is a significant negative correlation with a correlation coefficient of − 0.79.

The relationship between the thickness of the vadose zone and the EOL can be approximated as a quadratic function (R^2^ is close to 1; Fig. 6b); therefore, the fitting result is very reliable. According to the fitted curve, the increase in the thickness of the vadose zone would effectively extend the EOL of landfill^44^. As shown in Fig. 6c, the aquifer thickness has an approximately logarithmic relationship with the EOL; thus, increases in aquifer thickness will result in increases in the EOL^45^, with much greater growth occurring when the aquifer thickness increases from 5 to 10 m. These findings indicate that, when the aquifer thickness is less than 10 m, the EOL of the landfill will be significantly reduced. Correlation analysis showed that the thickness of the vadose zone and aquifer has a positive relationship with the EOL, with correlation coefficients of 0.99 and 0.72, respectively, indicating that the vadose zone thickness has a greater impact on EOL than the aquifer thickness.

Due to limitations of time and funds, the aging model adopted in this paper is relatively simple and can only roughly reflect the aging process of relevant units. At the same time, only three parameters are taken into account in the final analysis of EOL. Accordingly, the influence of landfill structure, initial permeability coefficient of impervious membrane, and initial vulnerability density on the EOL should be studied in future investigations.

### Model assumptions and application scope

the influence of pollutant discharge on groundwater under the leakage condition of hazardous waste landfill site was studied. In this case, the seepage rate is relatively small (< 10%) compared with the velocity of groundwater aquifer, so it can be assumed that the gradient change of groundwater caused by leachate leakage can be ignored. In addition, this study assumes that the vadose zone and aquifer are homogeneous isotropic media, the infiltration rate and concentration of leachate do not change with time, and the leachate and aquifer are uniformly mixed in the whole aquifer thickness. However, in the actual site, the underground medium is usually inhomogeneous. Since the leachate usually migrates in the upper part of the saturated aquifer after it seeps into the aquifer, the larger thickness is not considered. Only when the leakage volume is very large, the vertical mixing of the whole aquifer saturated thickness will occur.

Since the percolation of leachate may be a continuous process, it is reasonable to assume that the aquifer thickness is between 1–10 m and vertical mixing, that is, when pollutants migrate from the vadose zone to the aquifer, the influence of pollutant concentration on dilution and diffusion is fully considered, but the specific process is not discussed. In order to measure the uncertainty caused by these simplified assumptions, Monte Carlo framework is used to consider the randomness of model variables.

### Uncertainty analysis

In this study, the safety life of a specific HWL is evaluated. The characteristic pollutants are determined by the main waste received by the HWL and cannot represent all the HWLs. Then, for the evaluation of the aging process of the impermeable membrane, the main reference is other literatures, and the lack of on-site testing of the impermeable membrane samples may lead to the deviation of the prediction results. Therefore, when evaluating the safety life of a specific HWL, the characteristic pollutants should be determined according to the relevant conditions of HWL, and the impermeable membrane samples should be collected for testing, to accurately evaluate the EOL.

## Conclusion

As the geomembrane ages and the drainage system clogs, the leakage rate of leachate in HWLs will increase over time. Its environmental risk will also increase, and the HWL will reach its EOL. As for this case, its EOL was 385 a.

Based on different protection objects (i.e., Zn, inorganic fluorides, and Cr), calculations of the security life cycles were different (i.e., 625, 385, and 470 a, respectively). Therefore, inorganic fluorides should be regarded as key pollutants, and their leaching concentrations should be limited in order to reduce their health risks and increase the safety of the landfill life cycle.

Analyses on the limit values of leaching concentration, the thickness of vadose and aquifer zones indicated that the parameter of leaching concentrations was negatively related to EOL, while the thickness of the vadose and aquifer zones was positively related to EOL. Among them, the parameter with the greatest influence on EOL was vadose thickness, of which the coefficient of correlation was 0.99; the limit value of leaching concentrations and aquifer thickness had coefficients of correlation equal to − 0.79 and 0.72, respectively.
